# Promoting Angiogenesis Effect and Molecular Mechanism of Isopropyl Caffeate (KYZ), a Novel Metabolism-Derived Candidate Drug, Based on Integrated Network Pharmacology and Transgenic Zebrafish Models

**DOI:** 10.3389/fphar.2022.901460

**Published:** 2022-06-02

**Authors:** Haotian Kong, Songsong Wang, Yougang Zhang, Yangtengjiao Zhang, Qiuxia He, Rong Dong, Xiaohui Zheng, Kechun Liu, Liwen Han

**Affiliations:** ^1^ School of Pharmacy and Pharmaceutical Science, Shandong First Medical University and Shandong Academy of Medical Sciences, Jinan, China; ^2^ School of Basic Medical Sciences, Shandong University, Jinan, China; ^3^ Biology Institute, Qilu University of Technology (Shandong Academy of Sciences), Jinan, China; ^4^ Key Laboratory of Resource Biology and Biotechnology in Western China, Ministry of Education, The College of Life Sciences, Northwest University, Xi’an, China

**Keywords:** Ischemic diseases, Angiogenesis, Isopropyl Caffeate, Network Pharmacology, transgenic zebrafish, HUVECs

## Abstract

Aim of the study: Ischemic diseases have a huge impact on people’s health, which can cause blood supply blockage or restriction in specific tissues. Researchers must develop novel drugs with great efficacy and low toxicity for the prevention and treatment of such diseases. Isopropyl caffeic acid (KYZ) was one of the metabolites of caffeic acid *in vivo*. This study is to explore the protective effect and mechanism of KYZ on ischemic disease from the perspective of angiogenesis *in vivo* and *in vitro*, providing support for the treatment of ischemic diseases and the discovery of a new candidate drug. Methods: The network pharmacology and molecular docking were used to predict the targets of KYZ. In addition, the effects of KYZ on damaged and normal blood vessels were evaluated using the *Tg (fli1: EGFP)* transgenic zebrafish. The HUVECs model was used to study the effects of KYZ on proliferation, migration, and tube formation. The same dosage of caffeic acid (CA) was also administered *in vitro* and *in vivo* at the same time to assess the pharmacodynamic difference between the two compounds. Western Blot and ELISA methods were used to detect the expression of related target proteins. Results: The result from the network pharmacology indicated that the targets of KYZ were related to angiogenesis. It was also found that KYZ could repair the vascular damage induced by the PTK787 and promote the growth of subintestinal vessels in normal zebrafish. The result also indicated that KYZ’s angiogenic ability is better than the precursor compound CA. In HUVECs, KYZ could promote cell proliferation, migration, and tube formation. Further mechanistic study suggested that the KYZ could induce the release of VEGF factor in HUVECs, up-regulate the expression of VEGFR2, and activate the PI3K/AKT and MEK/ERK signaling pathways. Conclusions: These data show that KYZ may promote angiogenesis through VEGF, PI3K/AKT, and MEK/ERK signaling pathways, suggesting that KYZ exhibited great potential in the treatment of ischemic cardio-cerebrovascular diseases.

## Introduction

Ischemic disease caused by a blockage or restriction in blood supply to specific tissues or organs, such as ischemic stroke, ischemic heart disease, and critical limb, was extremely damaging to human health. The common pathological basis of ischemic disease was tissue damage and necrosis caused by sudden ischemia (Herrington et al., 2016; Severino et al., 2020; Zheng et al., 2020). The quality of the patient’s life has been seriously impaired by ischemic disease, presenting a high risk of disability and death. Therefore, it is of great significance to discover the new drugs for the treatment of ischemic diseases.

The current mainstream strategy for new drug discovery is a target-based structural modification, for instance, the modification of oridonin can improve its water solubility and anticancer activity (Xu et al., 2014). With the development of science and technology, multi-target-based strategies including bioinformatics and computer tools have been widely applied to the new drug discovery. Metabolites of chemical compounds are an important source for drug discovery and development. Many active metabolites make an important contribution to pharmacodynamics and then are listed as new drugs (Wang et al., 2015). The discovery of new therapeutic agents from metabolites has evolved into a novel drug discovery strategy. In terms of safety, drug discovery based on *in vivo* metabolism was considered to be more reliable. Traditional Chinese medicine had thousands of years of experience in the prevention and treatment of ischemic diseases. In recent years, many studies found that caffeic acid from popular medicines *Salvia miltiorrhiza Bge* have antioxidant, anti-inflammatory, and anticarcinogenic activity (Espindola et al., 2019)**.** However, some studies also showed that caffeic acid had a short half-life time in rats (Wu et al., 2015), which limited its usage in the clinic. In our previous study, we found that caffeic acid was metabolized to isopropylated metabolites in rats and this metabolite was named as isopropyl caffeate (KYZ). Based on this important discovery, we speculated that the pharmacological effect of caffeic acid may be performed by KYZ because it is naturally metabolized in the body, and KYZ may have theoretically better security than caffeic acid.

Generation of new blood vessels from preexisting vascular structures is called angiogenesis, which plays a significant role in many physiological and pathological conditions. In addition, angiogenesis is a primary adaptive pathophysiologic response during ischemic diseases and is deemed to be one of the key approaches to the treatment of ischemic diseases (Marti and Risau, 1999; Zhao et al., 2017). The pro-angiogenic factors including basic fibroblast growth factor (βFGF), vascular endothelial growth factor (VEGF), and transforming growth factor-beta (TGF-β) et al. help the body balance angiogenesis (Zhao et al., 2015). For the past several years, many angiogenesis models *in vivo* were established to study vascular development, among which the zebrafish are especially suited for the vascular system analysis.

Therefore, KYZ was synthesized chemically, and the pharmacological effect and mechanism responsible for KYZ angiogenesis activity were investigated. We assessed the pro-angiogenic effects of KYZ in zebrafish and human umbilical vein endothelial cell (HUVEC) models, by evaluating the reconstruction of damaged blood vessels and the normal blood vessel growth, as well as cell proliferation, migration and tube formation. The molecular mechanism responsible for the treatment of ischemic diseases by KYZ were also evaluated, providing support for pharmacological parameters of KYZ.

## Materials and Methods

### Chemicals and Reagents

The KYZ was synthesized chemically by Professor Xiaohui Zheng from Northwest University, China. According to the principle of TCM combinatorial formulations (Zhao et al., 2015), the KYZ was discovered and synthesized (Yang, 2018; Gao, 2019). Ethyl 3-aminobenzoate methanesulfonate salt (MS-222, Cat: 886-86-2, >98% purity), phenltiocarbamide (PTU, Cat: 103-85-5), and methylcellulose (Cat: 9004-67-5) were purchased from Sigma-Aldrich. Danhong Injection (DHI, Cat: 1901101062) was purchased from Shandong Buchang Pharmaceutical Co., Ltd. Dimethyl sulfoxide (DMSO) was purchased from Sangon Biotech Co., Ltd., (Shanghai, China). Trypsin (Cat:1930154), Fetal Bovine Serum (FBS, Cat: 42F0266K), Penicillin-Streptomycin (Cat: 15140122), DMEM-H basic (Cat: 8119410), MEM and NEAA (Cat: 2028909) were purchased from Gibco company.

### Zebrafish Maintenance and Embryo Collection

The *Tg (fli1: EGFP)* transgenic zebrafish line was provided by Engineering Research Center of Zebrafish Models for Human Diseases and Drug Screening of Shandong Province, and was raised separately by gender, and fed with live shrimp every day. The zebrafish were maintained at 28.5°C with a 14 h/10 h light/dark cycle in an automatic breeding system (ESEN, Beijing, China). Before the experiment, the male and female zebrafish at a ratio of 1:1 were placed in the breeding tank, and the male and female zebrafish were separated. The separated partition was removed before the next day’s light. The fertilized eggs were retrieved and were washed with fresh water and were cultured in incubator at 28.5°C.

### The Target Prediction of Isopropyl Caffeic Acid

Swiss Target Prediction database (https://www.swisstargetprediction.ch) was an algorithm used for the prediction of targets of small molecule compounds (David et al., 2014) and was applied to our study to predict the potential targets of KYZ. KEGG pathway enrichment analysis was performed using the predicted targets in the DAVID database (https://david.ncifcrf.gov). The results of the KEGG enrichment analysis were visualized by the online tool (https://www.omicshare.com). We docked the core, and downloaded the protein structure in the PDB (http://www.rcsb.org/) database. Discovery Studio 2.5 software was used for docking analysis between protein targets from the selected pathway with KYZ, and the protein targets with high scores were verified in zebrafish and HUVEC experiments.

### Mortality of Zebrafish Treated With Isopropyl Caffeic Acid

Healthy *Tg (fli1: EGFP)* transgenic line zebrafish embryos after 24 h post-fertilization (hpf) was selected under a fluorescence microscope (SZX16, Olympus, Tokyo, Japan). Pronase-E (Solarbio, Shanghai, China) was used to remove the egg membrane. The zebrafish larvae were randomly placed into 24-well microplates, 15 each well. KYZ was dissolved in DMSO and then was diluted to different concentrations with fresh fish water. The final volume of the solution in each well was 2 ml. The control group was treated with 0.5% DMSO. After KYZ treatment, the culture plate was placed in the incubator. The number of death larvae were counted at 48 hpf, and LC_50_ was calculated by Graphpad prism software. The experiment was repeated three times.

### Effect of Isopropyl Caffeic Acid on Intersegmental Vascular Injury in Zebrafish

To study the effect of KYZ on injured vessels of zebrafish, PTK787 (Cat: SML2498, Sigma, United States) was used to induce intersegmental vascular injury model (Wood et al., 2000). The experiment was performed in 24-well microplates with 12 larvae each well. KYZ and CA were diluted to the required concentrations with fresh water, and the control group was treated with the same volume of 0.5% DMSO (*v*/*v*). Danhong injection (DHI) was used as a positive control. In this experiment, 24 hpf larvae were co-treated with PTK787 and KYZ for 24 h according to the method previously reported (Liao et al., 2019). Larvae were anesthetized with 0.02% tricaine methanesulfonate and were photographed under a fluorescence stereomicroscope (AXIO Zoom. V16, ZEISS, Oberkochen, Germany). The length of ISVs of larvae were determined by Image-Pro Plus software (Media Cybernetics, Bethesda, MD, United States).

### Effect of Isopropyl Caffeic Acid on Normal Subintestinal Vessels of Zebrafish

To study the effect of KYZ on normal subintestinal vessel of zebrafish, the PTU was added to the culture medium to inhibit the accumulation of melanin in zebrafish. Larvae at 72 hpf were treated with different concentrations of KYZ and CA for 24 h at 28.5°C. The abdominal SIVs of zebrafish were observed and were photographed using a fluorescence stereomicroscope after being anesthetized with 0.02% tricaine methanesulfonate. The distance of SIVs was measured by Image-Pro Plus software. All experiments were repeated three times.

### Cell Culture

Human umbilical vein endothelial cells (HUVECs) were purchased from Beijing Dingguo Changsheng Biotechnology Co., Ltd and were maintained in DMEM-H basic medium with 10% FBS, 1% Penicillin-Streptomycin, and 1% MEM NEAA at 37°C.

### Cell Proliferation Assay

The HUVECs were cultured in 96-well microplates (2.5 × 10^3^/100 µl). After 12 h, the culture medium was removed and was replaced with medium without FBS. Then, HUVECs were starved for 12 h. The HUVECs were then treated with different concentrations of KYZ and CA that dissolved in 0.5% FBS medium, respectively. The control group was treated with the same volume of DMSO. VEGF growth factor at a concentration of 20 ng/ml was used as positive control. After 24 h incubation, the HUVECs viability was measured by CCK-8 analysis according to the manufacturer’s instructions.

### Wound Healing Assay of HUVEC

Wound healing assays were performed as previously described with minor modifications (Schinwald et al., 2012). The HUVECs were cultured in 6-well microplates. When the cells covered 90% of the bottom of microplate, pipette tip was used to draw a straight line in each well. Then the culture medium was removed, and the microplate was rinsed with PBS three times to remove the floating cell debris. After starvation for 12 h, HUVEC were treated with KYZ and CA for 24 h, respectively. The image of wound healing was photographed under a microscope (IX16, Olympus, Tokyo, Japan) at 0 and 24 h. Finally, the area of wound healing was measured by ImageJ software.

### Transwell Migration Assay

The migration ability of HUVECs was assessed in the Transwell. The culture medium with different concentrations of KYZ and CA was placed in the bottom wells of the chamber, HUVECs (5 × 10^3^) were then loaded in the upper chamber. The plates were incubated in a humidified incubator with 37°C and 5% CO_2_. After 4 h, the non-migrated cells in the upper chamber were removed with a cotton swab, and the chamber was washed with PBS three times. HUVECs were fixed with 4% paraformaldehyde for 20 min, and were stained with 0.1% crystal violet for 20 min. The images were photographed by microscope (IX16, Olympus, Tokyo, Japan). ImageJ software was used to quantify the migration cell on the lower surface of the member, and three fields were chosen randomly for each chamber.

### Tube Formation Assay

The HUVECs were cultured in 6-well microplates. After starvation for 12 h, HUVECs were treated with KYZ and CA for 24 h, respectively. Then, HUVECs were dissociated by 0.25% trypsin were plated in 96-well microplates coated with Matrigel. After 8 h, the tubes were randomly selected from three visual fields to take pictures. For the quantification of the tube formation, the junctures with at least three tubes converged were calculated.

### ELISA

The HUVECs were cultured in 6-well microplate at a density of 2 × 10^5^ per well. When covered 70–80% of the bottom of the well, HUVECs were incubated with a serum-free medium and then were treated with different concentrations of KYZ. At 24 h and 48 h, 50 µl cultured medium was collected and secreted VEGF level was analyzed by the ELISA kit (Cat: DVE00, R&D Systems, United States) according to the manufacturer’s instructions. The experiment was repeated three times.

### Western Blot

HUVECs were treated with different concentrations of KYZ for 24 h. In the inhibition assay, the cells were pre-treated with LY294002 or U0126 for 30 min before the treatment with KYZ. The culture medium was removed, and the cells were washed three times with ice-cold PBS before collection using ice-cold RIPA buffer (Cat: P0013B, Beyotime, China). The cell disruption was operated with an ultrasonic cell disruption system and the homogenate was centrifuged at 11000 rpm/min for 10 min. The supernatant was collected and the protein concentration was determined using BCA kit (Cat: P0012, Beyotime, China). Then the supernatant and SDS-PAGF (5 × ) were degenerated for 10 min at 100°C. The proteins were electrically transferred to the 0.22-μm NC membrane and blocked by TBST (0.1% Tween-20) with 5% protease-free BSA (Cat: ST025, Beyotime, China). The following antibodies were used: GAPDH (1: 10,000, Cat: BA2913, Boster Biological Technology Co. Ltd., China), VEGF-R2 (1:1,000, Cat: #9698, Cell Signalling Technology, United States), ERK1/2 (1:1,000, Cat: #4695, Cell Signalling Technology, United States), p-ERK1/2, (1:1,000, Cat: #4370, Cell Signalling Technology, United States), MEK1/2 (1:1,000, Cat: #8727, Cell Signalling Technology, United States), AKT (1:1,000, Cat: #4691, Cell Signalling Technology, United States), p-AKT (1:1,000, Cat: #4060, Cell SignallingTechnology, United States), goat anti-mouse IgG-HRP (1:10,000, Cat: abs20001, Absin Bioscience, Inc., Shanghai, China), goat anti-rabbit IgG-HRP (1: 10,000, Cat: abs20002, Absin Bioscience, Inc., Shanghai, China). The following inhibitor was used: LY294002 (Cat: HY-10108, MedChem Express), U0126 (Cat: HY-12031A, MedChem Express).

### Data and Statistical Analyzed

All data were analyzed using SPSS 25.0 or GraphPad Prism 8.0 software. The Kolmogorov-Smirnov test was used to test the normality of data. One-way ANOVA was used and data were expressed as mean ± standard error (SEM). A *p*-value less than 0.05 was considered as significant.

## Results

### The Network Prediction and Molecular Docking of Isopropyl Caffeic Acid

**FIGURE 1:**
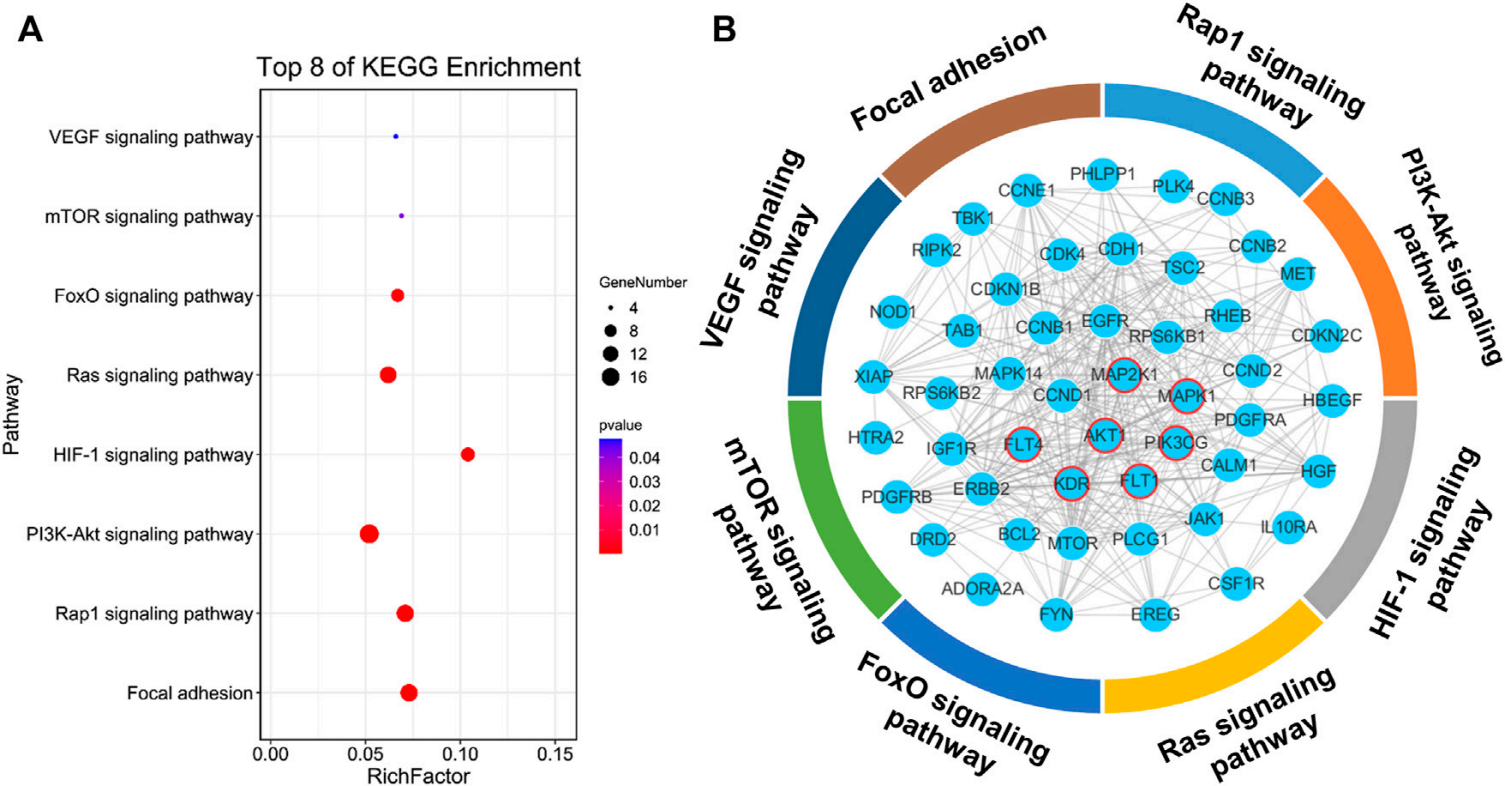
Target prediction of KYZ. **(A)** KYZ’s targets were obtained using the Swiss Target Prediction and then the targets were put into the DAVID database for KEGG analysis. Eight pathways associated with vascular disease in KEGG enrichment analysis was demonstrated. **(B)** The protein interaction network between eight vascular disease-related pathways, the proteins circled in red are the direct target of KYZ, other proteins were enriched in these pathways.

**FIGURE 2:**
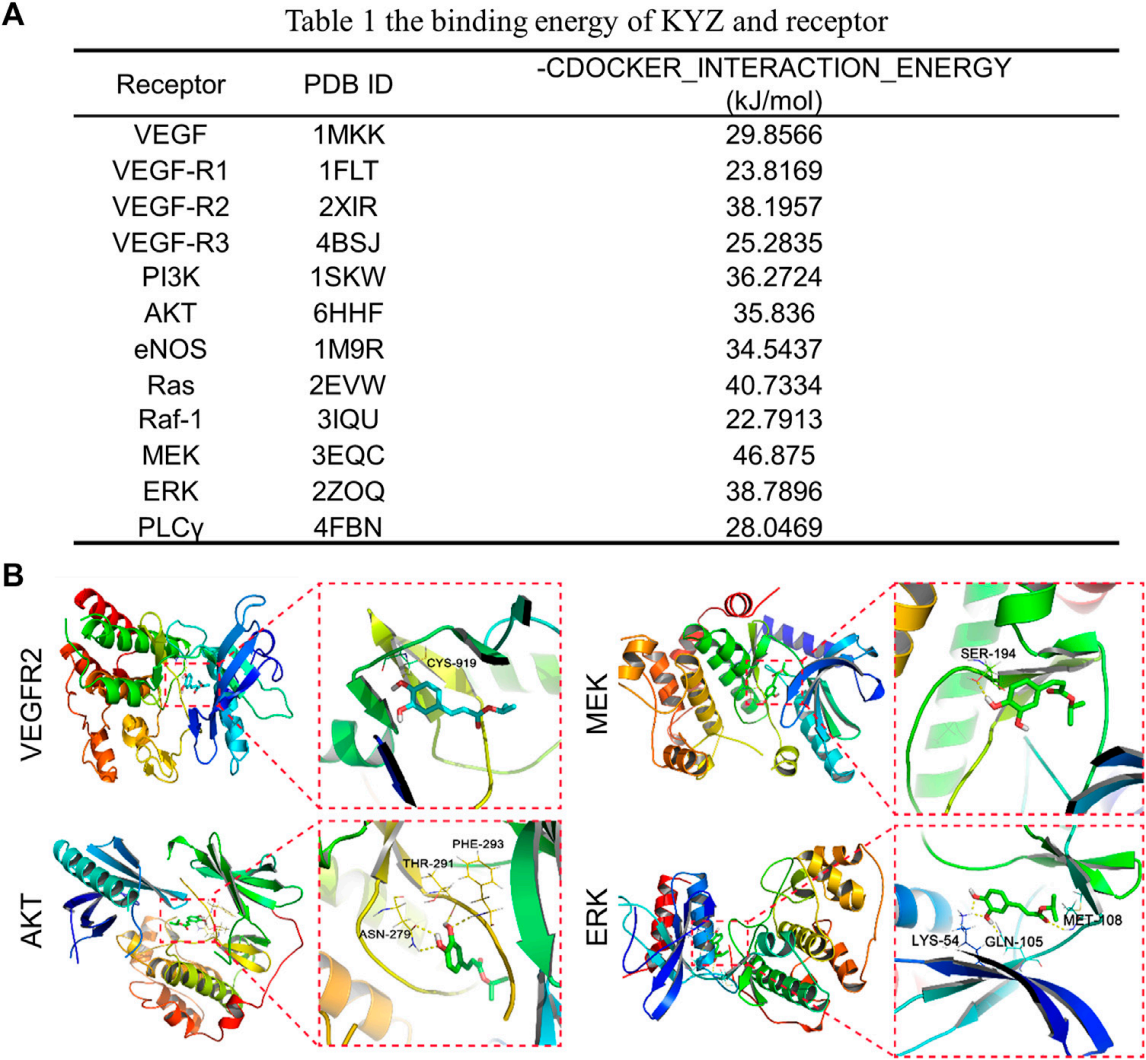
**(A)** The binding energy of KYZ with the predicted protein receptors. The lower binding energy indicates the higher affinity of KYZ with the target protein. **(B)** Docking analysis of KYZ with proteins VEGFR2, AKT, MEK1/2, ERK1/2 visualized using Pymol software.

In the target prediction experiment, 100 potential KYZ targets were obtained using the Swiss Target Prediction database. The predicted targets of KYZ were then put into the DAVID database for KEGG analysis. According to the result of KEGG pathway enrichment analysis, 70 pathways were enriched in total, with 38 pathways statistically significant and eight pathways related to vascular disease, as shown in Figure 1A. The protein interaction network between these eight pathways was visualized in Figure 1B. It’s worth noting that some targets associated with promoting angiogenesis were included from our current investigation. Therefore, protein targets from VEGF, Ras, PI3K/Akt and MAPK pathways were selected to conduct molecular docking analysis, and the result is shown in Figure 2A. The binding energy of reverse molecular docking analysis indicated that VEGFR2, PI3K, AKT, MEK1/2, and ERK1/2 play important roles in promoting angiogenesis by KYZ treatment.

### Isopropyl Caffeic Acid Reduced Inhibition of Angiogenesis in Zebrafish

**FIGURE 3:**
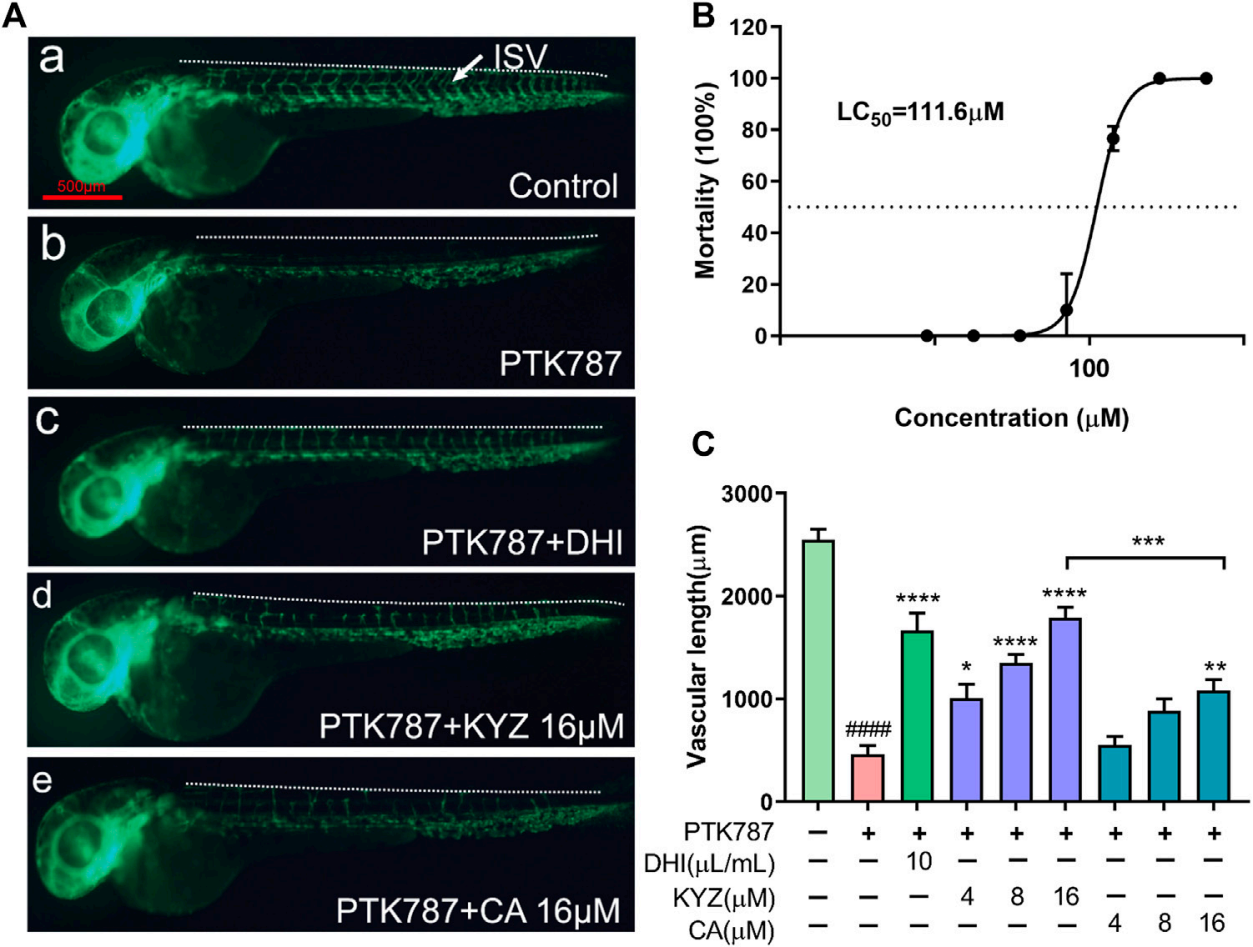
Repair effects of different concentrations of KYZ and CA on PTK787-induced ISVs injury in zebrafish. **(A)** KYZ treatment rescued the zebrafish ISVs injury induced by PTK787. Lateral view of the zebrafish treated with PTK787, PTK787 + DHI, PTK787 + KYZ (4,8,16 μM), and PTK787 + CA (4,8,16 μM) for 24 h, respectively. DHI was used as positive control, and the white arrow indicates the intersegmental vessel (ISV). **(B)** The 24 hpf *Tg (fli1: EGFP)* line zebrafish were treated with different concentration KYZ. After treatment for 24 h, the number of death larvae was counted and converted into percentages, and LC_50_ was calculated by Graphpad prism 8.0. software. **(C)** Quantitative analysis of the total length of ISV in zebrafish by KYZ and CA treatment, and the data are expressed as mean ± SEM. One-way ANOVA was used for statistical analysis (n = 12). *p < 0.05, **p < 0.01, ***p < 0.0001, ****p < 0.0001, ####*p* < 0.0001 compared with control group. Scale bars is 500 μm.

The *Tg (fli1: EGFP)* transgenic zebrafish model was used to evaluate the promoting angiogenesis activity of KYZ. The results indicated that the LC_50_ of KYZ in 48 hpf zebrafish was 111.6 μM, as shown in Figure 3B. Angiogenesis of zebrafish intersegmental vessels in model group was almost completely inhibited after treatment with PTK787 at a concentration of 0.175 μg/ml. DHI could significantly reverse the ISVs injury with a recovery rate of more than 70% (*p* < 0.001). The treatment with KYZ could significantly rescue ISVs formation induced by PTK787 treatment in a dose dependent manner (*p* < 0.0001) (Figures 3A,C). However, the CA treatment at a concentration of 16 μM could reverse the PTK787 induced angiogenesis inhibition in zebrafish (*p* < 0.01). Interestingly, compared with CA treatment group, the KYZ treatment had better rescued capacity at a concentration of 16 μM (*p* < 0.001), indicating that KYZ had better effect than CA in reversing vascular damage in zebrafish (Figures 3A,C).

### Isopropyl Caffeic Acid Promoted Subintestinal Vessels Growth in Zebrafish

**FIGURE 4:**
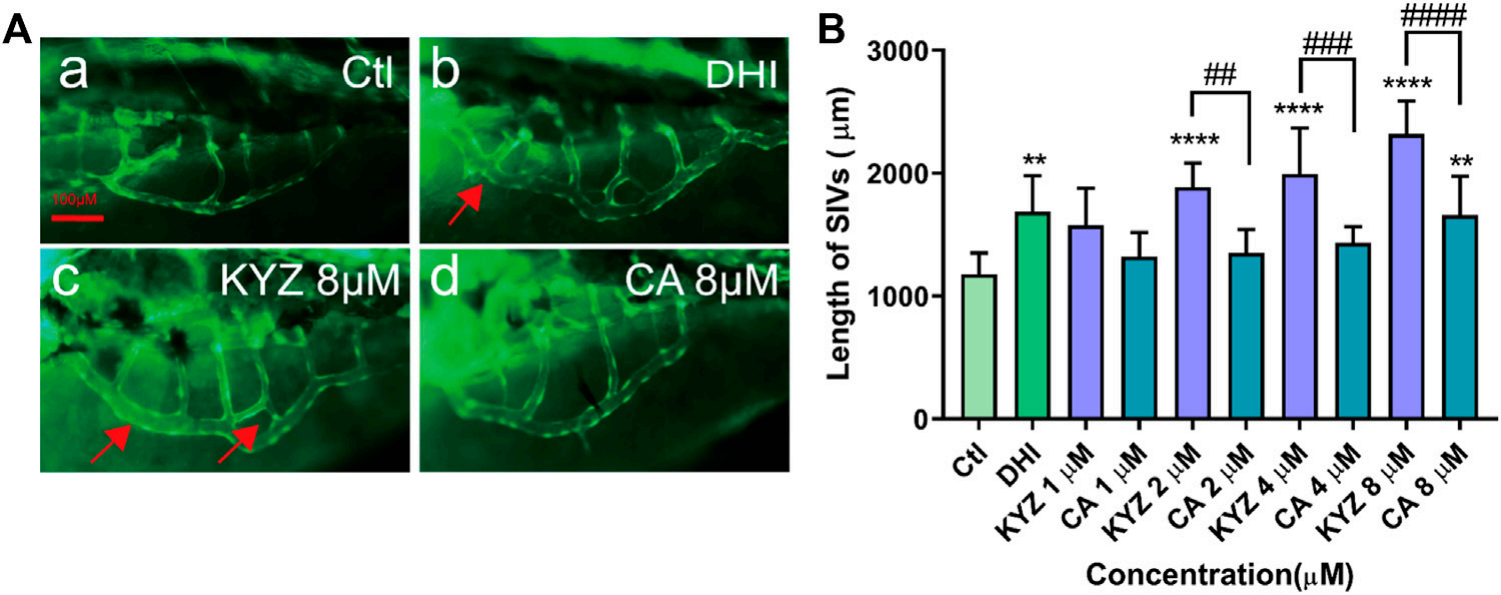
KYZ and CA treatment promoted SIVs growth in zebrafish. **(A)** The red arrow indicates cross-linking and budding of SIVs. The 48 hpf zebrafish embryos were treated with KYZ and CA at concentrations of 1, 2, 4, 8 μM for 24 h, and the result showed that there was a growth of SIVs observed. DHI was used as a positive control. Scale bar is 100 μm. **(B)** Quantitative analysis of the total length of the SIVs in zebrafish after treatment with KYZ, the data were expressed as mean ± SEM. One-way ANOVA was used for statistical analysis (*n* = 12). **p < 0.05,****p < 0.0001 vs. control group. ##*p* < 0.01,###*p* < 0.001,####*p* < 0.0001, KYZ vs CA.

The effects of promoting the growth of normal zebrafish subintestinal vessels by KYZ treatment was also evaluated. Figure 4A showed a smooth basket-like structure of normal SIVs in zebrafish. Both DHI and KYZ treatment could significantly promote the growth of SIVs in zebrafish, by exhibiting the increase in blood vessel diameter and length, cross-linking of blood vessels, and the formation of sprouting. Compared with the control group, the total length of SIVs in the 2 μM KYZ treated group significantly increased (*p* < 0.0001). Following the increasing concentrations of KYZ, the growth promoting effect of KYZ in SIVs was more obvious, indicating that KYZ promoted the SIVs in a dose dependent manner. For the CA treatment group, only 8 μM CA treated zebrafish showed an obvious increase in length of SIVs (*p* < 0.01), indicating that the KYZ had better activity than CA in the promotion of zebrafish SIVs (*p* < 0.0001), as indicated in Figure 4B.

### Isopropyl Caffeic Acid Promoted Proliferation and Motility of HUVECs

**FIGURE 5:**
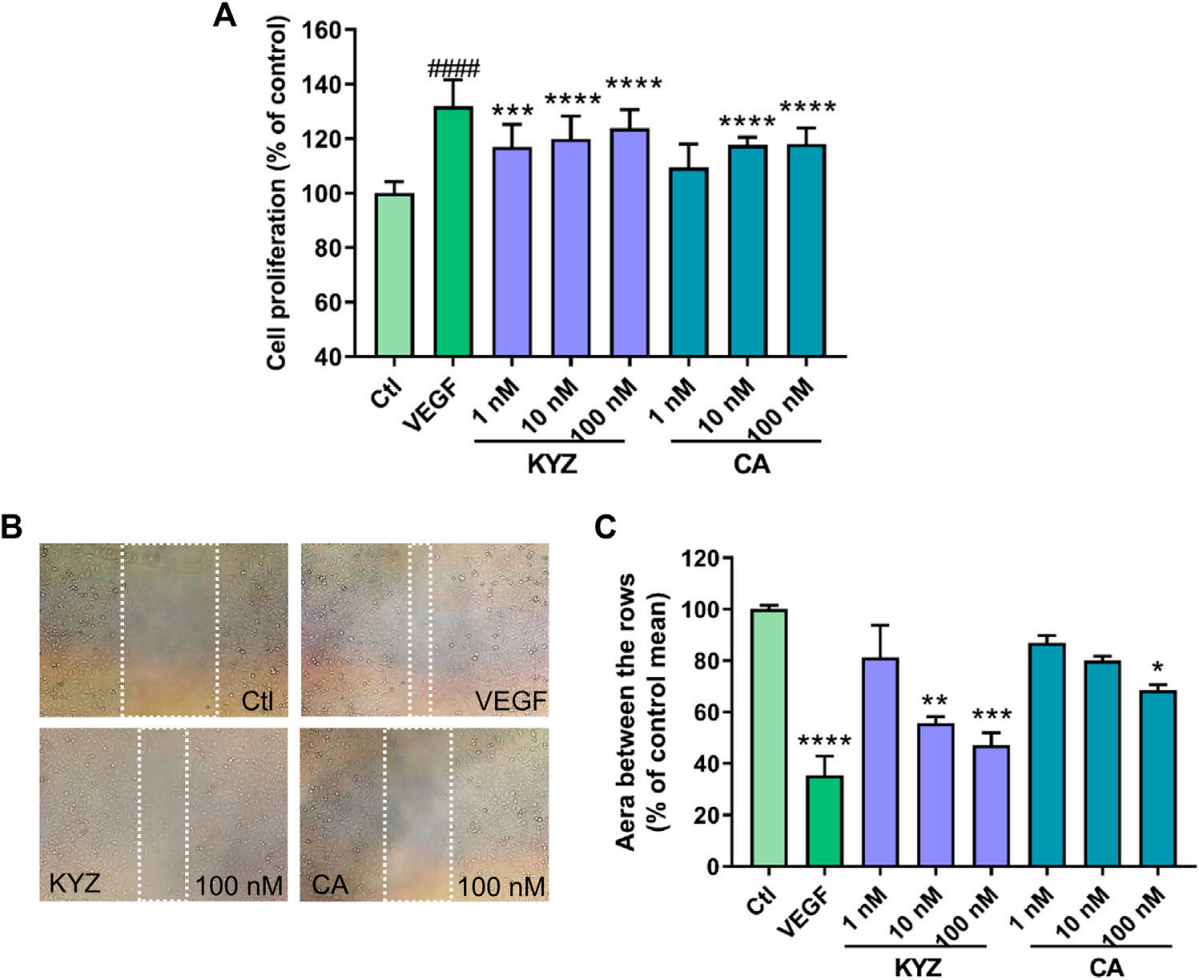
KYZ and CA treatment promoted HUVECs proliferation and enhanced repair wound ability **(A)** HUVECs were cultured in 96-well microplates (2.5 × 10^3^ cells/100 µl). After treatment for 12 h, the culture medium was removed and was replaced with medium without FBA for starvation, then HUVECs were treated with KYZ and CA. After treatment for 24 h, cell proliferation was measured by CCK-8 assay. **(B)** HUVECs were scratched using pipette tips to induce the wound. The white area indicated the size of the wound after treatment with the KYZ and CA for 24 h. The photomicrographs were shown at ×10 magnification. **(C)** Quantitative analysis of the scratch area of HUVECs (the area between two white line) by ImageJ software. The scratch area of the control group was defined as 100%. Results were shown as mean ± SEM. One-way ANOVA was used for statistical analysis. *p < 0.05,**p < 0.01,***p < 0.001,****p < 0.0001 vs. control.

The KYZ treatment could significantly promote the HUVECs proliferation in a dose dependent manner. Compared to the control group, the cells proliferation rate in 100 nM KYZ treated group were 24% (*p <* 0.0001). However, the cells proliferation rate in the 100 nM CA treated group was 18% (*p* < 0.0001) (Figure 5A). Overall, there was no significant difference between the KYZ treatment groups and CA treatment groups in promoting cell proliferation. Relative growth rate of the positive group treated with VEGF was 31% (*p* < 0.0001), compared to the control group (Figure 5A). The effects of KYZ and CA on the healing ability of HUVECs were investigated through wound healing experiments. The results are shown in Figures 5B,C. In wound healing assay, the area of scratch wound healing was measured at 0 and 24 h after the HUVECs were wounded. Compared with the control group, HUVECs treated with KYZ for 24 h could significantly increase the wound healing of HUVECs in a dose dependent manner and the healing rate of 100 nM KYZ was 52.87% (*p <* 0.001). However, only high concentration of CA treated HUVECs were capable of improving wound healing, and the healing rate was only 31.54% (*p* < 0.05). VEGF treatment could increase wound closure with a healing rate of 64.59% (*p* < 0.0001).

### Isopropyl Caffeic Acid Enhanced the Migration Ability of HUVECs

**FIGURE 6:**
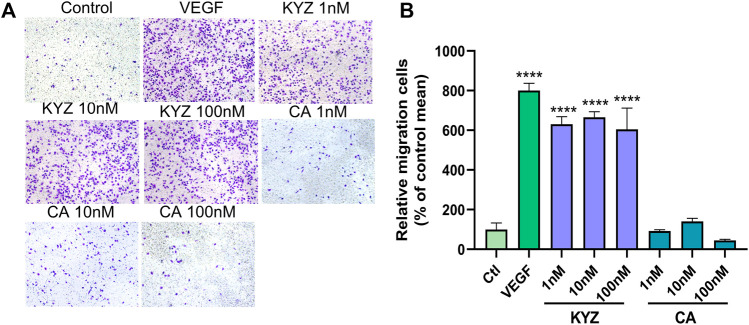
The KYZ treatment enhanced the migration of HUVECs. **(A)** The stained cells crossed the polycarbonate membrane by treatment with KYZ and CA for 24 h. The VEGF (20 ng/ml) was used as a positive control. The photomicrographs were shown at × 10 magnification. **(B)** Statistical results of cell migration in each group. The results were expressed as a percentage of the mean to the control group (mean ± SEM). Statistical analysis was performed using a one-way analysis of variance, *n* = 3. **p < 0.01,****p < 0.0001 vs. control.

The effect of KYZ and CA on HUVEC motility was also investigated. The number of cells that passed the polycarbonate membrane was used as an indicator in this essay, as shown in Figure 6. Compared with the control group, treatment with KYZ for 4 h could significantly induce the migration of HUVECs from the upper chamber to the lower chamber (*p* < 0.001). However, CA treatment did not influence the migration of the HUVECs.

### Isopropyl Caffeic Acid Enhanced the Tube Formation of HUVECs

**FIGURE 7:**
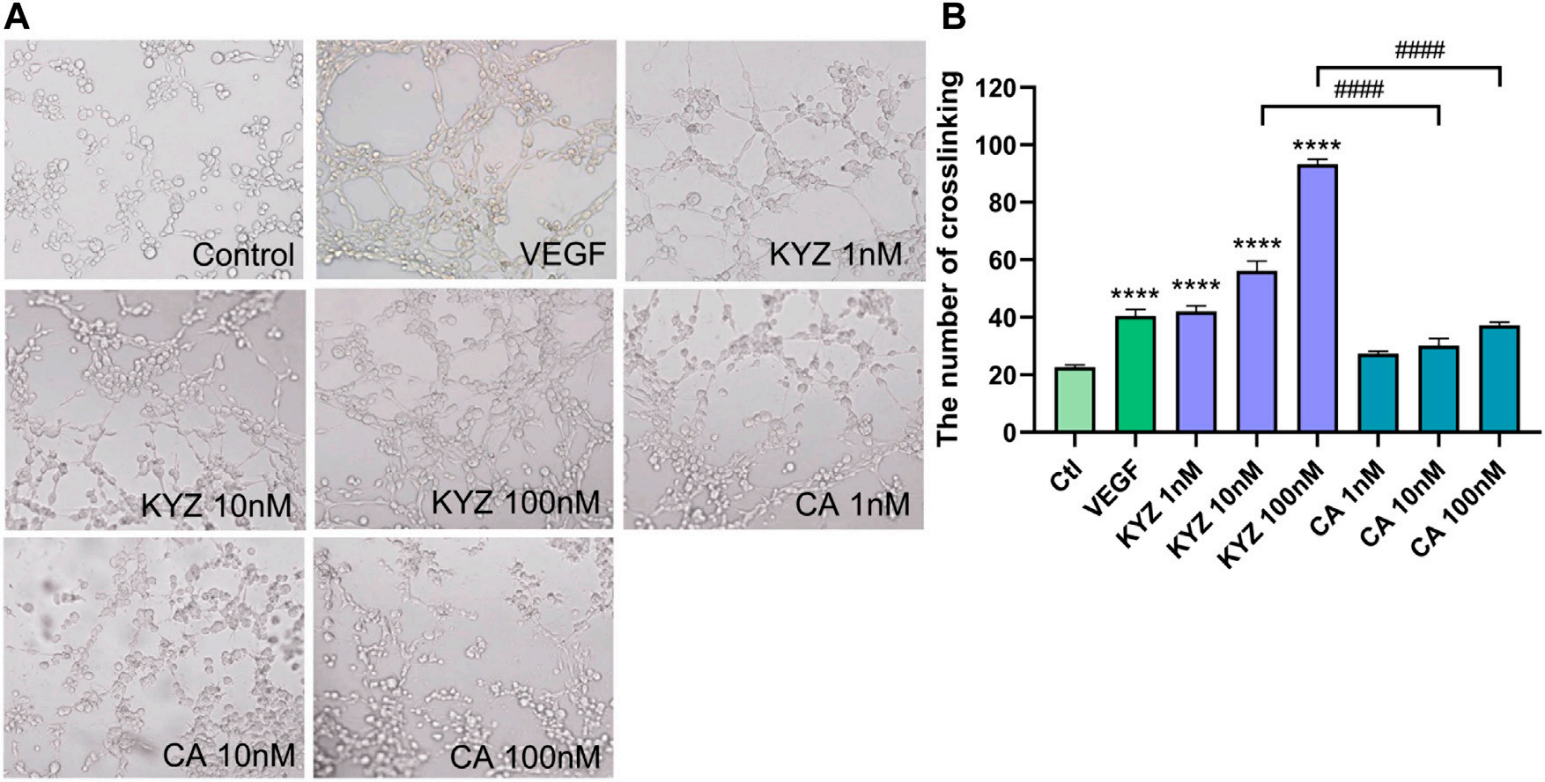
The KYZ treatment enhanced tube formation of HUVECs. **(A)** The HUVECs were pretreated with difference concentration KYZ and CA for 24 h, then the cells were cultured on the Matrigel. After treatment for 8 h, the tube structure was photographed, and the photomicrographs were shown at × 4 magnification, and the number of tube junctures were counted. **(B)** Quantitative analysis the number of tube junctures in each group by ImageJ software. The results were shown as mean ± SEM. Statistical analysis was performed using a one-way analysis of variance, *n* = 3. ***p < 0.001,****p < 0.0001 vs. control.

The effect of KYZ and CA on the tube structure of HUVECs was also evaluated following the culture of HUVECs on Matrigel with low serum medium for 8 h. The result showed that there were only a few tubes formed in the control group. After KYZ treatment, the tube network of HUVECs gradually formed (Figure 7A). Compared with the control group, the KYZ treatment group formed more branch points in HUVECs, reaching the maximum value at a concentration of 100 nM (*p* < 0.0001). However, CA treatment at a concentration of 100 nM only formed a small number of branch points (Figure 7B). It demonstrated that KYZ treatment could induce HUVECs to form a wider capillary network on the Matrigel.

### Isopropyl Caffeic Acid Enhanced VEGF Secretion and the VEGFR2 Expression in HUVECs

**FIGURE 8:**
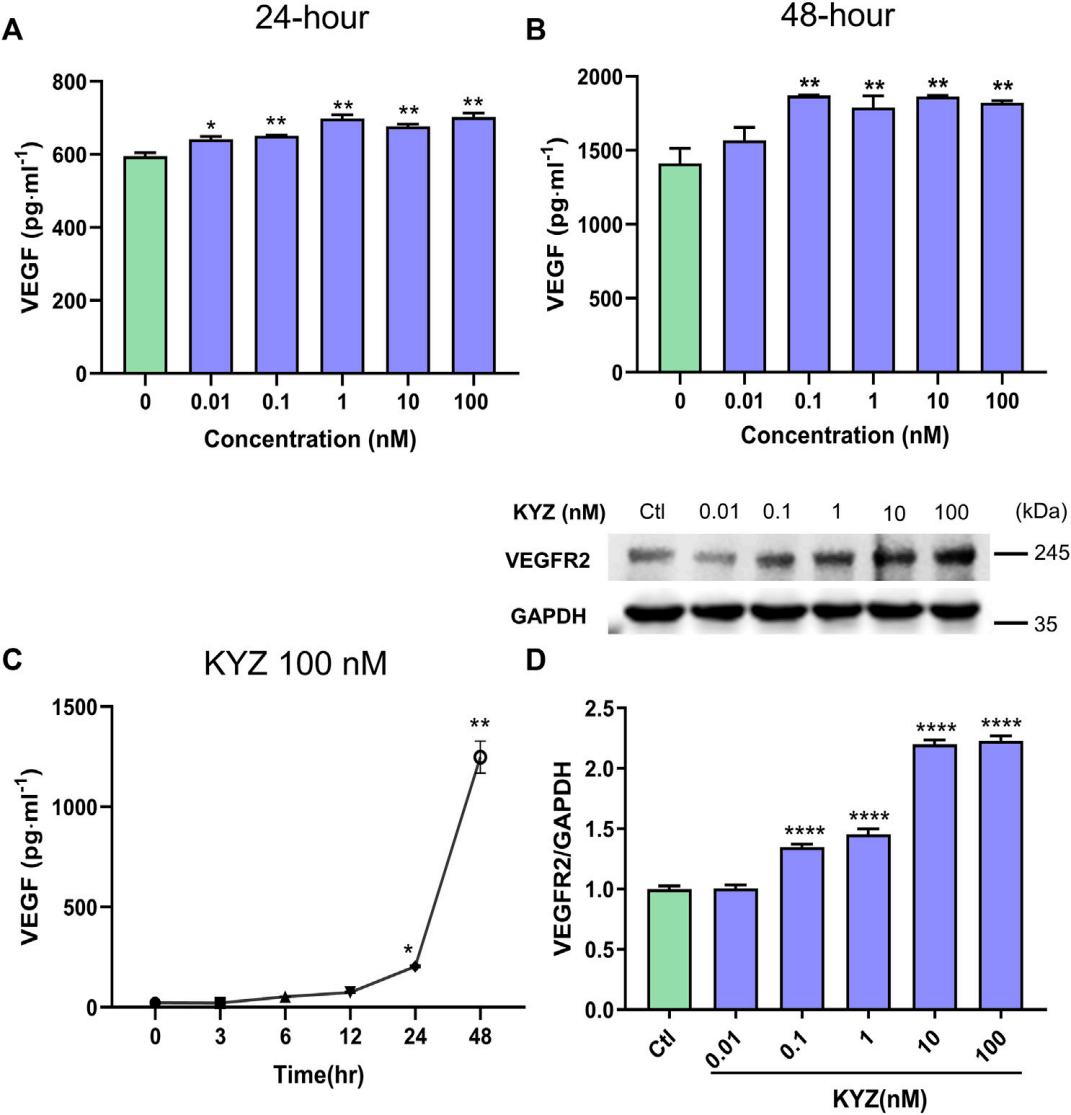
KYZ treatment increased the VEGF secretion and VEGFR2 expression in HUVECs. **(A–B)** HUVECs were cultured in 6-well microplate and were treated with different concentrations KYZ for 48 h, the total volume of culture medium was 5 ml. 50 µl culture medium was collected at 24 and 48 h, respectively. VEGF level was analysed by ELISA assay. **(C)** the HUVECs were treated with KYZ (100 nM) for 3, 6, 12, 24, 48 h, then the medium was collected and was analysed for VEGF secretion level by ELISA **(D)** the VEGFR2 expression was determined by western blotting and gray value of the result was analysed by ImageJ software. The data were expressed as mean ± SEM (*n* = 3). One-way ANOVA was used for statistical analysis. *p < 0.05,**p < 0.01,***p < 0.001,****p < 0.0001 vs control.

The binding of VEGFR2 with VEGF activates intracellular signaling pathways that lead to the growth of vascular endothelial cells and to promotion of angiogenesis (Peach et al., 2018). By combining the results of KEGG pathway enrichment analysis and molecular docking analysis, expression levels of proteins VEGF and VEGFR were evaluated. The ELISA assay was used to determine the expression level of VEGF secretion in the culture medium following KYZ treatment. The results showed that treatment with KYZ for 24 and 48 h could increase the expression level of VEGF in culture medium (*p* < 0.01), as shown in Figures 8A–C. Meanwhile, the expression level of VEGFR2 was significantly increased by the KYZ treatment at concentrations of 0.1 nM (*p* < 0.0001). These findings showed that KYZ might promote angiogenesis by increasing VEGF secretion and promoting VEGFR2 protein expression in HUVECs.

### Isopropyl Caffeic Acid Increased the Protein Expression of AKT, MEK1/2 and ERK1/2

**FIGURE 9:**
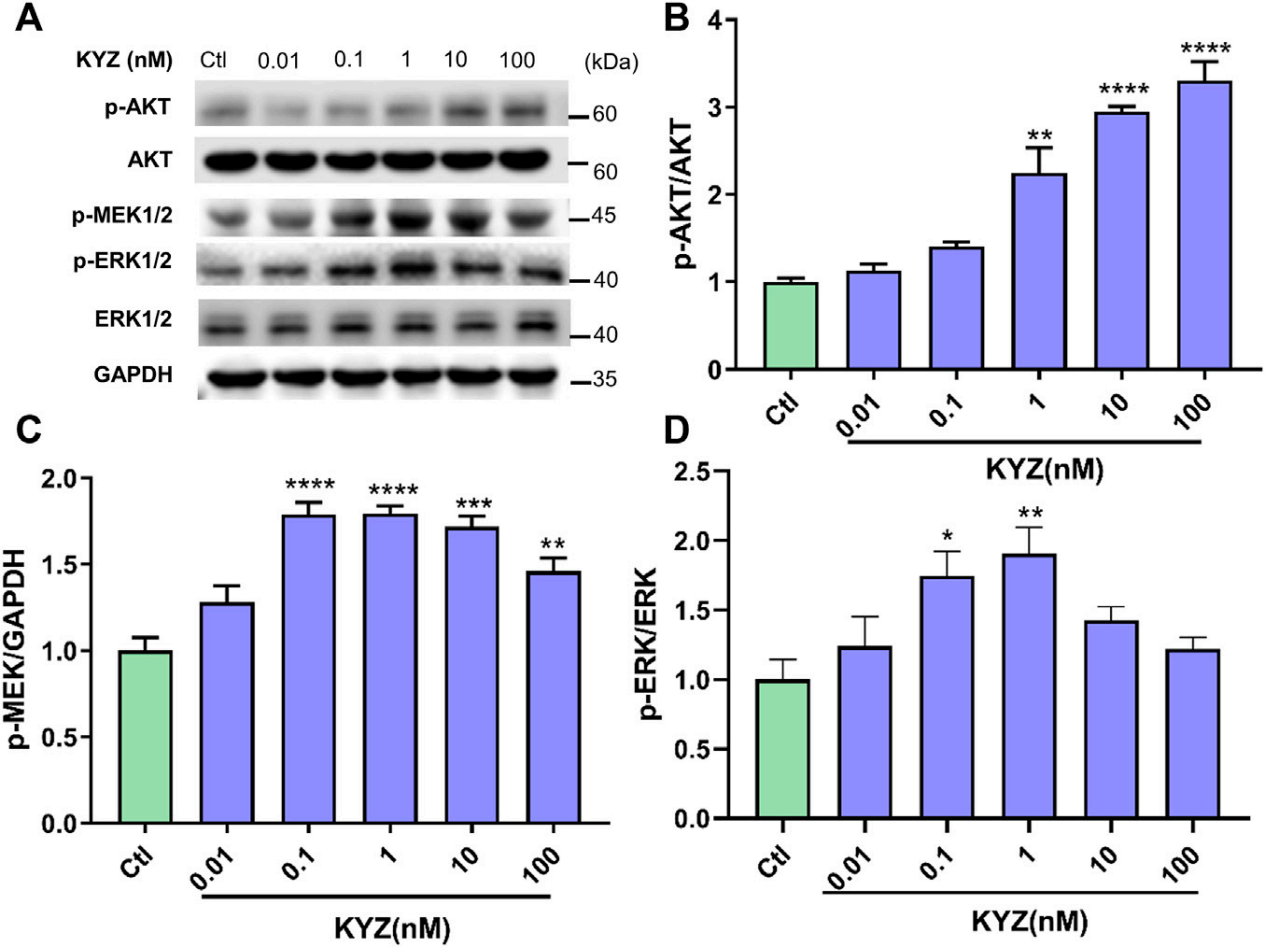
KYZ treatment increased the phosphorylation levels of AKT and MEK1/2 and ERK1/2 in HUVECs. The HUVECs were treated with different concentrations (0.01-100 nM) for 24 h. **(A)** The protein expressions of p-MEK1/2, p-ERK1/2, ERK, p-AKT and AKT. **(B)** The protein expressions ratio of p-AKT to AKT. **(C)** The protein expressions ratio of p-MEK to GAPDH. **(D)** The protein expressions ratio of p-ERK to ERK. The protein expressions were determined by western blotting and gray value were analysed by ImageJ software. The data were expressed as mean ± SEM (n = 3). One-way ANOVA was used for statistical analysis. *p < 0.05,**p < 0.01,***p < 0.001,****p < 0.0001 vs control.

AKT, MEK1/2 and ERK1/2 were important targets in our target prediction of KYZ. The phosphorylation level of protein AKT in HUVECs was increased in a dose dependent manner after treatment with different concentrations of KYZ for 24 h, while the total AKT protein level did not change, as shown in Figure 9. In addition, in the MEK1/2/ERK1/2 pathway, the phosphorylation levels of proteins MEK1/2 and ERK1/2 could be significantly up-regulated after treatment with different concentrations of KYZ.

### Effect of the Inhibitor on PI3K/AKT and MEK1/2/ERK1/2 Pathway Expression

**FIGURE 10:**
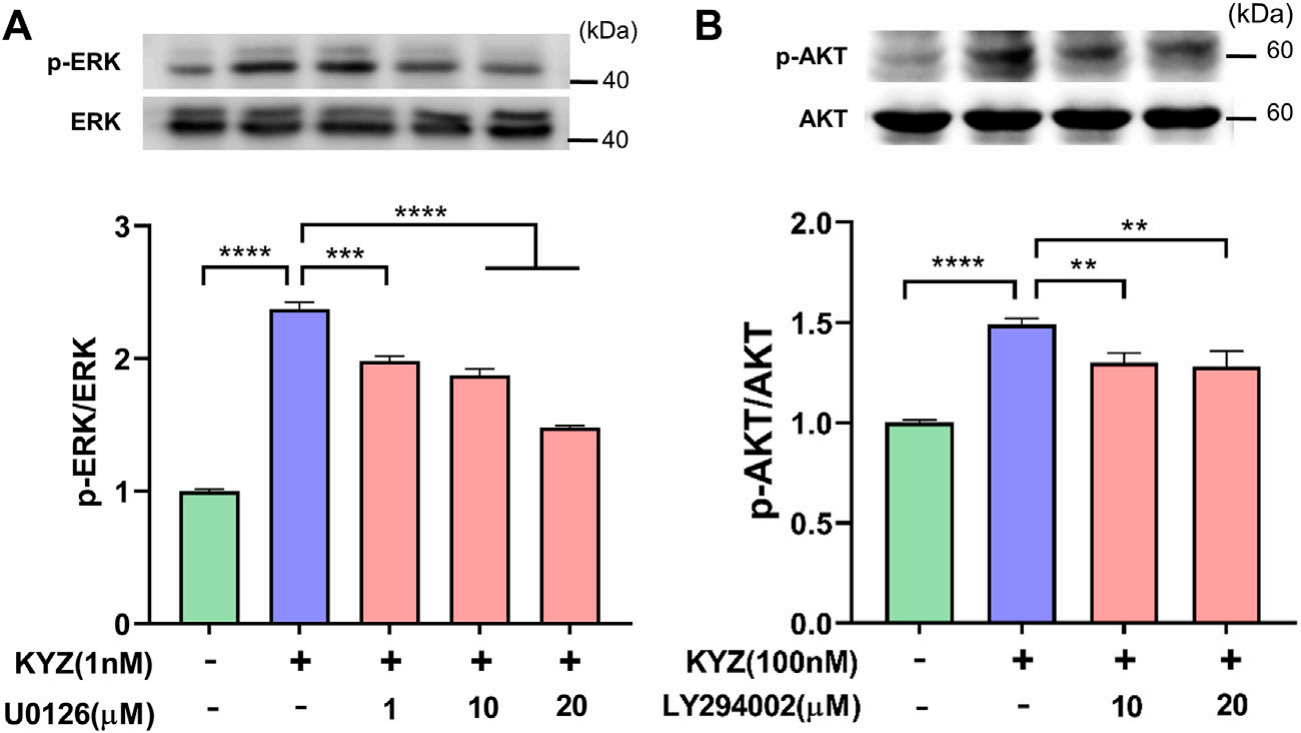
Effect of PI3K inhibitor LY29402 and MEK1/2 inhibitor U0126 on the promoting angiogenesis activity of KYZ. **(A)** The HUVECs were pre-treated with PI3K inhibitor LY294002 and MEK1/2 inhibitor U0126 for 30 min, following by KYZ treatment for 24 h. **(B)** the gray value of p-Akt and p-ERK were calculated by ImageJ software and expressed as mean ± SEM, **p < 0.01,***p < 0.001,****p < 0.0001 vs. control.

To further prove that the PI3K/AKT and MEK1/2/ERK1/2 pathways are related to pro-angiogenesis process of KYZ *in vitro*. Inhibitors LY294002 and U0126 were used to pretreat the cells for 30 min, respectively. The results showed that LY294002 treatment significantly inhibited the expression level of p-AKT in a dose dependent manner (Figure 10B). Similarly, it was also found that U0126 treatment could decrease the expression level of p-ERK (Figure 10A). This suggested that the PI3K/Akt and MEK1/2/ERK1/2 pathways were activated during the process of KYZ induced angiogenesis *in vitro.*

## Discussion

In this study, we proposed a strategy to fast validate and to confirm the pharmacological activity of KYZ, one of the metabolites of caffeic acid, based on the network target prediction and zebrafish models. Traditional drug screening often requires a large amount of manpower and financial resources, as well as the consumption of a large number of samples. In some instances, the finding and better understanding of active metabolites are pivotal in the drug discovery process, due to the reason that pharmacologically active metabolites generated by the metabolic transformation of xenobiotic substances have great chance to be responsible for therapeutic response of drugs (Kumar and Surapaneni, 2001; Fura, 2006). In practice, many drugs that prescribed in clinical produce pharmacological metabolites or the corresponding pharmacologically active metabolites are developed as new drugs, including simavastatin, atorvastatin, fexofenadine et al. (Fura, 2006). In previous studies, we found that caffeic acid, an active constituent of Salvia miltiorrhiza Bge, was metabolized to KYZ in rat, and KYZ was mainly existed in heart and brain tissue. More importantly, our data suggested that KYZ significantly improved cerebral blood flow, reduced the cerebral infarct volume, caused vasodilation, and improved vascular endothelial injury in mice, which was a highly potential new therapeutic compound for the treatment of Cardiovascular disease. However, as a new potential therapeutic chemical compound, the pharmacological characteristics and targets of KYZ are still unclear.

Computer virtual screening can effectively improve the accuracy of pharmacological mechanism studies, and can reduce the need for research space in laboratory experiments, therefore simulating the interaction between compounds and target proteins in the human body (Kanica and Manoj kumar, 2019). Swiss Target Prediction is a web-based tool that can map the potential targets of small molecules based on the similarity between the chemical molecules, enabling us to understand the bioactivity of small molecule deeply (Gfeller et al., 2014; Daina et al., 2019). In our study, 100 potential targets of KYZ were obtained in the Swiss Target Prediction experiment following by the KEGG analysis, in which 70 pathways were enriched and 38 in 70 pathways were considered statistically significant. These 38 pathways are mainly related to signal transduction, cell growth and death, and endocrine system. The result of literature survey revealed that two pathways, including PI3K and MAPK pathways, were significant transduction pathways involved in angiogenesis. In the molecular docking analysis, binding interactions of the KYZ with five protein targets including VEGFR2, PI3K, AKT, MEK1/2, ERK1/2 showed the minimum binding free energy, suggesting high binding affinity. These five protein targets are highly engaged in angiogenesis (Shiojima et al., 2002), indicating that the function of KYZ was related to angiogenesis.

A large number of zebrafish models were established to understand the mechanisms behind diseases, especially in the field of biomedicine, toxicology, and biotechnology research (Alestrom et al., 2020). The zebrafish model, as an excellent model for studying vascular biology *in vivo*, has allowed us to make significant progress in understanding the molecular and morphological processes of angiogenesis during blood vessel development. In zebrafish model, any defects in vascular development can be readily visible through *in vivo* microscopic observations, providing an excellent whole animal model for the study of angiogenesis (Bahrami and Childs, 2018). It is important to note that analysis of vessel formation can be operated by the detection of growing cells and cellular compartments detection in vascular specific transgenic fluorophore expression zebrafish. Therefore, the *Tg (fli1: EGFP)* transgenic strain zebrafish was used for the activity of promoting angiogenesis by KYZ treatment.

**FIGURE 11:**
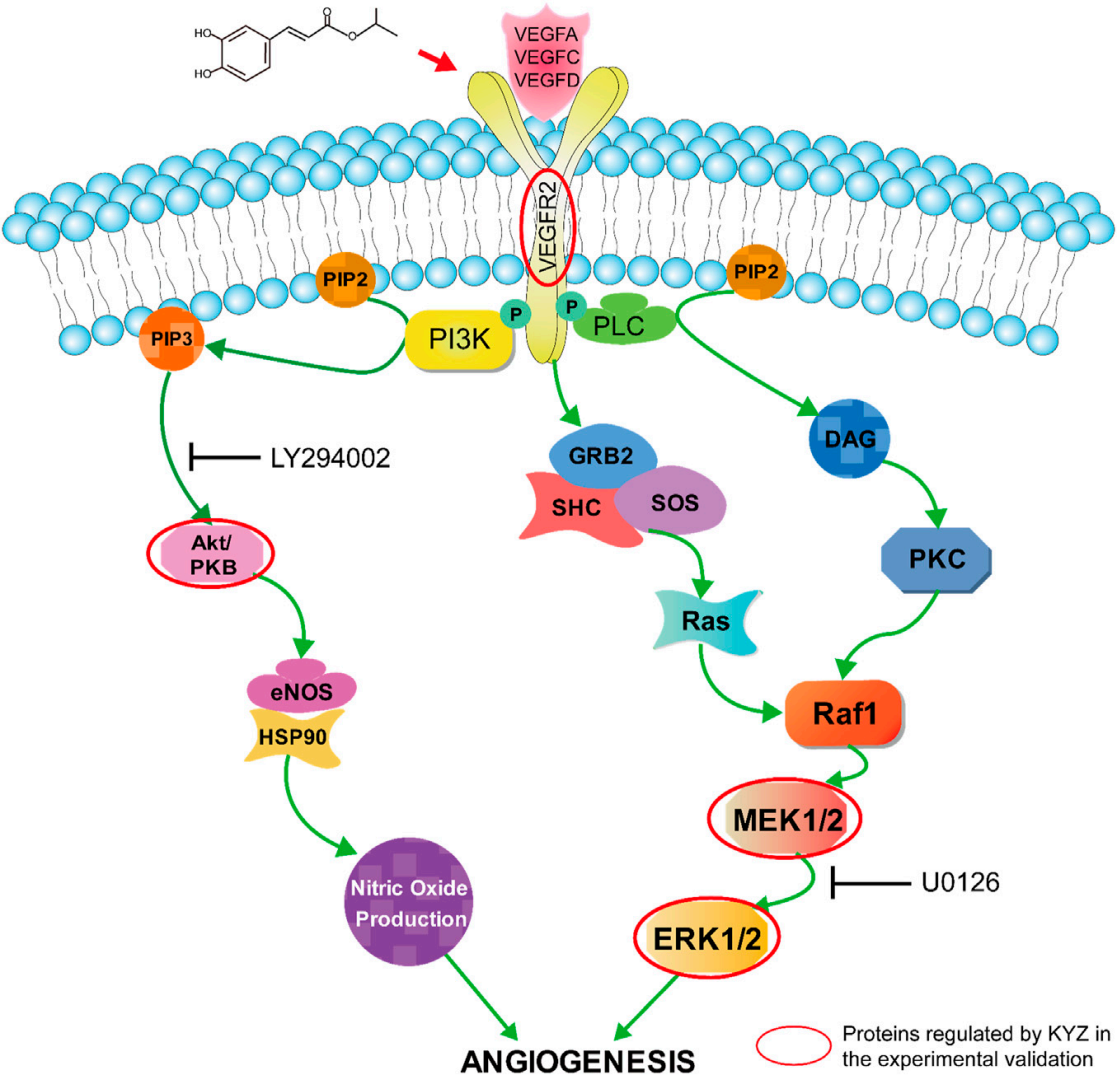
The possible mechanism responsible for promoting angiogenesis of KYZ, the proteins circled in red are the direct targets of KYZ.

Our data showed that KYZ was potentially effective drugs to treat ischemic disease *via* regulating angiogenesis. It has been proposed that angiogenesis has the function of tissue repair and regeneration when ischemia occurred, which is beneficial to functional recovery (Li et al., 2018). However, the process of angiogenesis was relatively slow, cannot supplying enough blood to ischemic tissues. Therefore, a novel approach to promoting angiogenesis by pharmacological stimulation has been proposed. PTK787 is an active inhibitor of VEGF-mediated angiogenesis (Wood et al., 2000; Bayliss et al., 2006). In our study, KYZ treatment could reverse the internode vessels injury induced by PTK787 treatment in zebrafish, indicating that KYZ may promote angiogenesis in zebrafish by increasing the VEGF secretion. Simultaneously, KYZ treatment could promote the growth of intestinal vein in zebrafish, confirming the pro-angiogenesis effect of KYZ *in vivo*. As a key regulator of pathogenic angiogenesis, VEGF secretion was significantly increased in HUVECs treated with KYZ for 24 and 48 h. Besides, VEGF could bind to vascular endothelial growth factor receptor 2, driving physiological and pathophysiological angiogenesis (Peach et al., 2018). The up-regulation of VEGFR2 was also observed under the challenge of KYZ in HUVECs. These data indicated that the angiogenesis promoting effect of KYZ is more likely to depend on VEGF/VEGFR-2 signaling pathways. In the endothelial cell lines, the binding of VEGF to VEGFR2 can activate the downstream MEK/ERK transduction pathway, finally resulting in angiogenesis (Zhou et al., 2017). In our study, the results indicated that KYZ could increase the phosphorylation levels of MEK and that the high phosphorylation levels of ERK was blocked after the treatment of inhibitors U0126 for 30 min, indicating that regulation of angiogenesis by KYZ is related to the MEK/ERK pathway. AKT is one of the downstream target proteins of VEGF/VEGFR-2 signaling pathway, and the binding of VEGF to VEGFR2 can also activate the PI3K, further promoting the phosphorylation of Akt, by which the angiogenesis is promoted (Shibuya et al., 2006). Simultaneously, the phosphorylation of Akt activate eNOS release and increase the production of NO, which is also highly associated with angiogenesis. Our result indicated that phosphorylation expression of AKT was increased after KYZ treatment and up-regulation of AKT phosphorylation level induced by KYZ treatment was reduced after the phosphorylation inhibitor LY29002 treatment in HUVECs, indicating that KYZ promoted angiogenesis through the PI3K/AKT pathway. The possible mechanism by which KYZ promote angiogenesis was shown in Figure 11.

Interestingly, some studies reported that the precursor caffeic acid can block VEGF expression, thus suppressing tumor angiogenesis (Jung et al., 2007; Izuta et al., 2009), which is inconsistent with our data that caffeic acid treatment could also promote angiogenesis in zebrafish. However, in the proliferation, migration, and tube formation experiment, CA treatment to HUVECs promoted proliferation and motility and did not enhance the migration ability and tube formation of HUVEC, thus, we speculated that the angiogenesis activity of CA may depend on proliferation and motility activity. Another reason is likely to be the problem with concentration of CA. The CA concentration applied in anti-angiogenesis effect studies were 15–200 μM, which was 2,000 times higher than the CA concentration used in our study. Therefore, we can deduce that high concentration CA exhibits anti-angiogenesis activity and lower concentration CA exhibits angiogenesis activity.

Several limitations of this study were considered in our future research. For example, further investigation about the promoting angiogenesis effect of KYZ will be carried out in rats and mice model. In addition, the toxicological assessment of KYZ will be operated *in vivo* and *in vitro* to guarantee the safety of KYZ.

## Conclusion

In present study, we developed an integrated strategy for rapid screening of pharmacological activities based on network pharmacology, molecular docking technology and models *in vivo* and *in vitro*. To the best of our knowledge, this is the first time that the potential novel drug KYZ has been shown to promote angiogenesis *in vitro* and *in vivo*. Furthermore, it has been discovered that KYZ is more effective in angiogenesis than the precursor molecule CA. It also reflects the complex metabolic synergistic effect of traditional Chinese medicine components *in vivo*. Zebrafish model and cell experiments were used to reveal the mechanism being responsible for promoting angiogenesis by KYZ treatment, and the result indicated that it was likely to be associated with the activation of VEGF, PI3K/Akt and MEK/ERK pathways. It also provided experimental evidence for KYZ’s research and development of innovative drugs for the prevention and treatment of ischemia.

## Data Availability

The original contributions presented in the study are included in the article/Supplementary Material, further inquiries can be directed to the corresponding authors.
