# Supplementary material for: Promoting Angiogenesis Effect and Molecular Mechanism of Isopropyl Caffeate (KYZ), a Novel Metabolism-Derived Candidate Drug, Based on Integrated Network Pharmacology and Transgenic Zebrafish Models
Source: Front Pharmacol. 2022 Jun 2;13:901460. doi: 10.3389/fphar.2022.901460 (PMC9201573; doi:10.3389/fphar.2022.901460)
Supplement: Supplementary file 1 [file DataSheet1.docx]

**Promoting** **angiogenesis effect and molecular mechanism of Isopropyl Caffeate (KYZ), a novel metabolism-derived** **candidate drug, based on integrated network pharmacology and transgenic zebrafish models**

**Haotian Kong^a,d,1^， Songsong Wang ^a,1^， Yougang Zhang^a^， Qiuxia He^b^， Rong Dong^a^， Xiaohui Zheng^c,*^， Kechun Liu^b,*^， Liwen Han^a,*^**

a. School of Pharmacy and Pharmaceutical Science, Shandong First Medical University and Shandong Academy of Medical Sciences, Jinan, China

b. Biology Institute of Shandong Academy of Sciences, Jinan, Shandong, China

c. College of Life Sciences, Northwest University, Xi’an, 710069, China

d. School of Basic Medical Sciences, Shandong University, Jinan, China

^1^ These authors have contributed equally to this work and share the first authorship.

*Corresponding author at:[zhengxh318@nwu.edu.cn](mailto:zhengxh318@nwu.edu.cn) (X.-H.Zheng),[hliukch@sdas.org(K.-C](mailto:hliukch@sdas.org(K.-C).Liu),[hanliwen@sdfmu.edu.cn(L.-W](mailto:hanliwen@sdfmu.edu.cn(L.-W). Han).

Abbreviation

| KYZ | Isopropyl caffeic acid |
| --- | --- |
| CA | caffeic acid |
| PTU | N-Phenylthiourea |
| DHI | Danhong Injection |
| PTK787 | Vatalanib dihydrochloride |
| FBS | Fetal Bovine Serum |
| hpf | hour post fertilization |
| ISV | intersegmental vascular |
| SIV | subintestinal vessel |
| CCK-8 | Cell Counting Kit-8 |


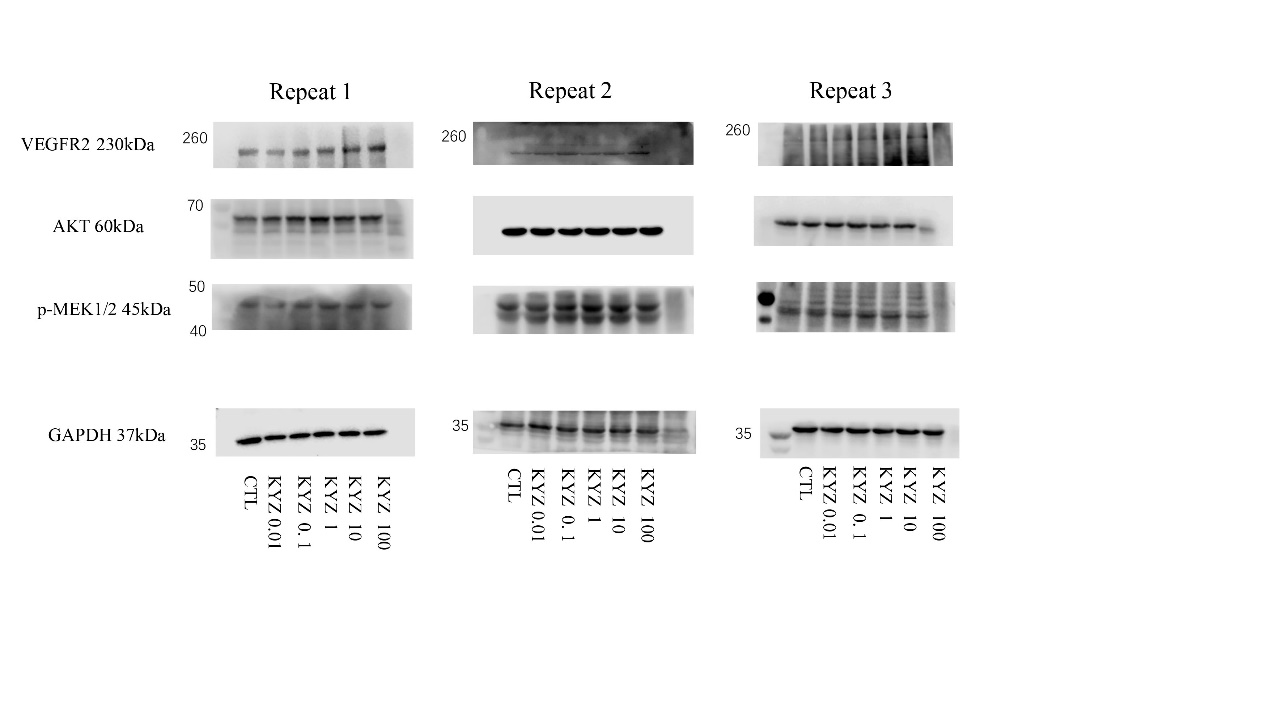


Fig.S1 The VEGFR2 and GAPDH represent western blot analysis shown in Fig.8

The AKT and p-MEK1/2 represent western blot analysis shown in Fig.9


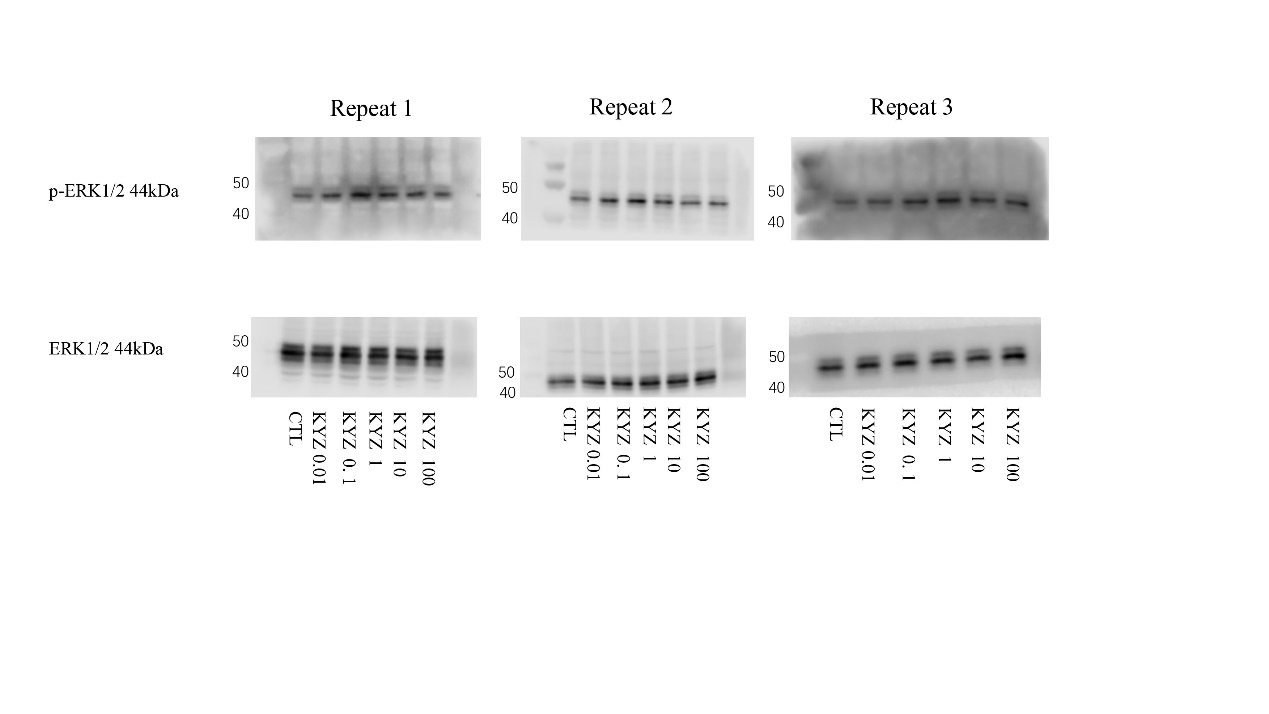


Fig.S2 The ERK1/2and p-ERK1/2 represent western blot analysis shown in Fig.9


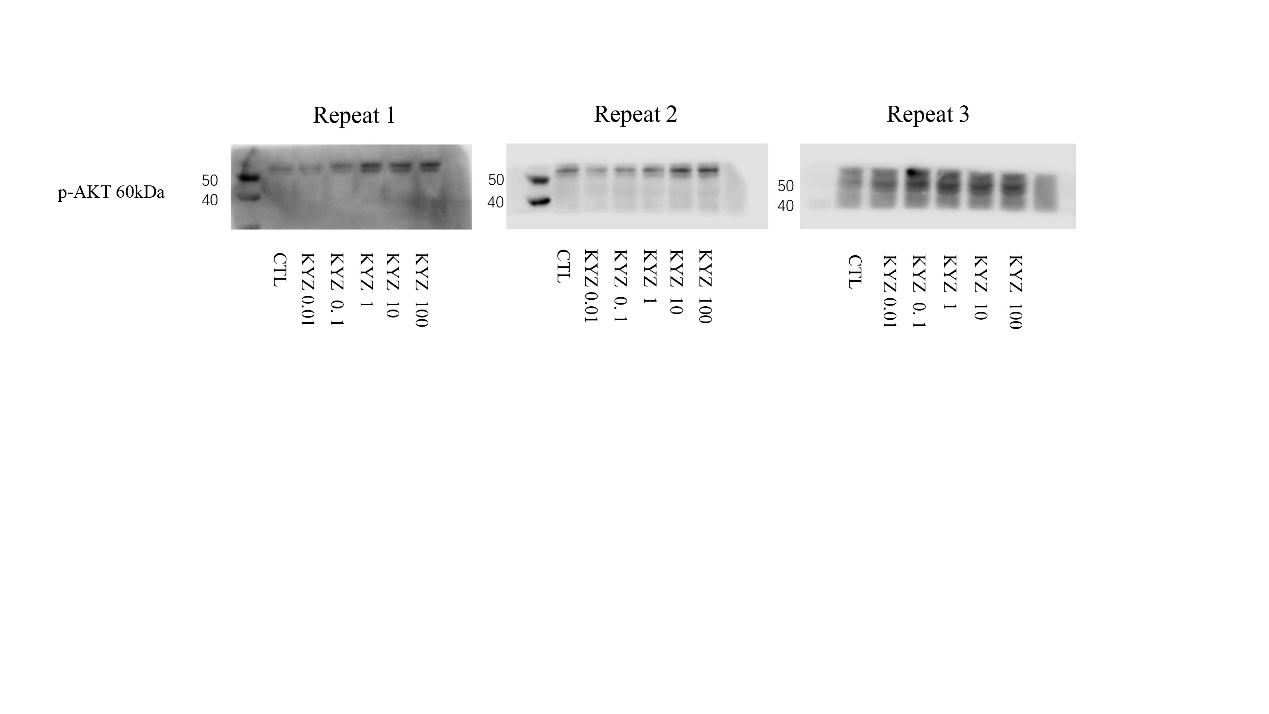


Fig.S3 The p-AKT represents western blot analysis shown in Fig.9


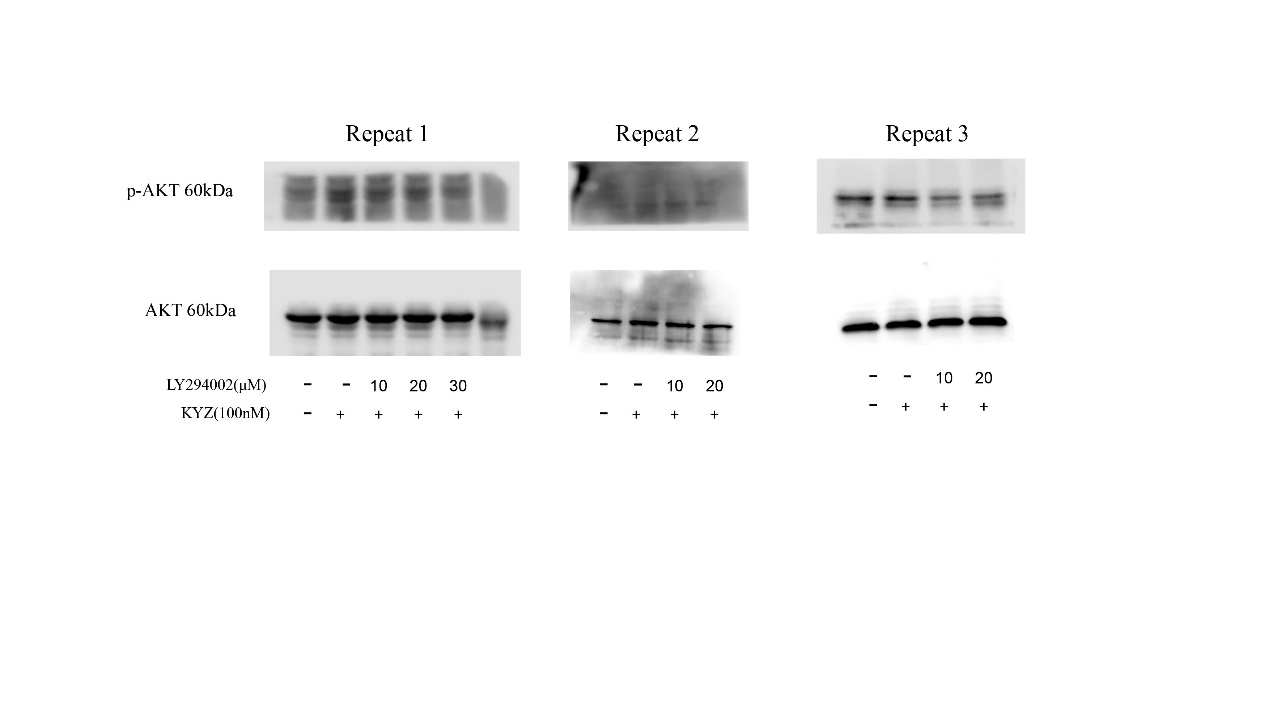


Fig.S4 The p-AKT and AKT represent western blot analysis shown in Fig.10


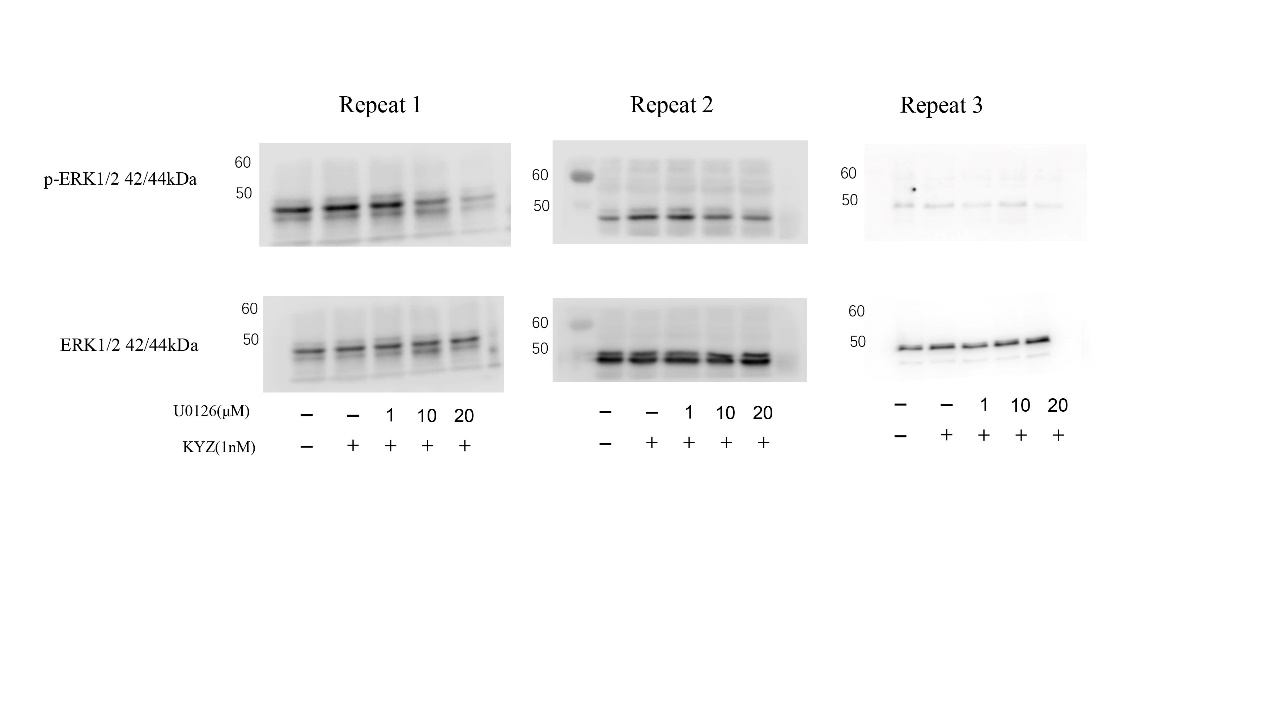


Fig.S5 The p-ERK1/2 and ERK1/2 represent western blot analysis shown in Fig.10
